# Pheromonal bile acid 3-ketopetromyzonol sulfate primes the neuroendocrine system in sea lamprey

**DOI:** 10.1186/1471-2202-14-11

**Published:** 2013-01-20

**Authors:** Yu-Wen Chung-Davidson, Huiyong Wang, Michael J Siefkes, Mara B Bryan, Hong Wu, Nicholas S Johnson, Weiming Li

**Affiliations:** 1 Department of Fisheries and Wildlife, Michigan State University, 13 Natural Resources Building, 480 Wilson Road, East Lansing, MI, 48824, USA; 2 Present address: Great Lakes Fishery Commission, 2100 Commonwealth Blvd., Suite 100, Ann Arbor, MI, 48105, USA; 3 Present address: Energy Biosciences Institute, University of California, 130 Calvin Laboratory, MC 5230, Berkeley, CA, 94720, USA; 4 Present address: Department of Microbiology & Immunology, School of Medicine, Emory University, Rollins Research Center G214, 201 Dowman Drive, Atlanta, Georgia, 30322, USA; 5 Present address: USGS, Great Lakes Science Center, Hammond Bay Biological Station, 11188 Ray Road, Millersburg, MI, 49759, USA

**Keywords:** Pheromone, Priming, HPG axis, GnRH, Steroid, Sexual dimorphism

## Abstract

**Background:**

Vertebrate pheromones are known to prime the endocrine system, especially the hypothalamic-pituitary-gonadal (HPG) axis. However, no known pheromone molecule has been shown to modulate directly the synthesis or release of gonadotropin releasing hormone (GnRH), the main regulator of the HPG axis. We selected sea lamprey (*Petromyzon marinus*) as a model system to determine whether a single pheromone component alters the output of GnRH.

Sea lamprey male sex pheromones contain a main component, 7α, 12α, 24-trihydroxy-5α-cholan-3-one 24-sulfate (3 keto-petromyzonol sulfate or 3kPZS), which has been shown to modulate behaviors of mature females. Through a series of experiments, we tested the hypothesis that 3kPZS modulates both synthesis and release of GnRH, and subsequently, HPG output in immature sea lamprey.

**Results:**

The results showed that natural male pheromone mixtures induced differential steroid responses but facilitated sexual maturation in both sexes of immature animals (χ^2^ = 5.042, dF = 1, p < 0.05). Exposure to 3kPZS increased plasma 15α-hydroxyprogesterone (15α-P) concentrations (one-way ANOVA, p < 0.05) and brain gene expressions (genes examined: three lamprey (l) GnRH-I transcripts, lGnRH-III, *Jun* and Jun N-terminal kinase (*JNK*); one-way ANOVA, p < 0.05), but did not alter the number of GnRH neurons in the hypothalamus in immature animals. In addition, 3kPZS treatments increased lGnRH peptide concentrations in the forebrain and modulated their levels in plasma. Overall, 3kPZS modulation of HPG axis is more pronounced in immature males than in females.

**Conclusions:**

We conclude that a single male pheromone component primes the HPG axis in immature sea lamprey in a sexually dimorphic manner.

## Background

The term “pheromones” was first introduced by Karlson and Lüscher (1959) to describe the substances involved in chemical communication among conspecifics [1]. Pheromones can be classified as releasers, primers, or more recently, signalers and modulators [2-6]. These functions are not mutually exclusive. Primer pheromones are exemplified by their effects on the onset of puberty, the length of estrous cycles in females, the success or failure of pregnancy, and shifts in hormone levels in mice [6]. Male pheromones have a direct impact on female sexual desire, menstrual cycles and ovulation in humans [7]. About 20% of women who have smelled male underarm secretions have an advanced onset of next luteinizing hormone (LH) pulse [8]. These primer pheromones are all putative pheromones since their chemical structures have yet to be determined.

Sex pheromones exert priming effects via the HPG axis that links environmental inputs to reproductive outputs [3]. Pheromone extracts or pheromone components have been shown to affect gonadotropin (GTH) or LH surge in many species [6,9,10]. However, no known pheromone component has been shown to directly induce GnRH release [9-12]. In fish, most studies of GnRH or GTH release have focused on seasonal changes and very few studies pay attention to the daily cycle or pulsatility of GnRH or GTH.

Sea lamprey provides an advantageous vertebrate model to examine the mechanisms by which a pheromone component primes the HPG axis since lamprey GnRH peptides are well characterized [13], and several pheromone molecules have been identified in this species [14-17]. Sea lamprey occupy a key position close to the root of the vertebrate phylogenetic tree [18,19]. They develop through three distinct life stages [20,21]. Larval sea lamprey spends several years in burrows as benthic filter feeders in the stream. After going through metamorphosis [22], the resulting parasitic juveniles enter large lakes or the ocean to feed on host fish. After 1.5 to 2 years, the adults cease feeding in the early spring and migrate into rivers to spawn and then die [20,21]. For parasitic sea lamprey, sexual maturation is halted until they cease feeding and begin an upstream spawning migration [23]. It is not clear how lGnRH-I and -III regulate the final maturation and whether there is a differential expression of hormones or prehormones during this stage. Males arrive at the spawning ground first, build a nest, and complete the final sexual maturation. Mature males release sex pheromones containing 3kPZS through the gills at the onset of spermiation [24]. It is known that 3kPZS stimulates olfactory receptor neurons in the olfactory epithelium [25], and induces upstream movement and searching behavior toward the nest in ovulatory females [14,26-28]. Downstream from the nests, immature animals are also exposed to 3kPZS, the possible effects of which have not been examined.

We hypothesized that 3kPZS exerts priming effects by altering the HPG output in immature sea lamprey. To test this hypothesis, we first examined the effect of natural pheromone mixtures on sexual maturation of immature lamprey. We then examined the dose response and the time course of synthesized 3kPZS on plasma 15α-P concentrations, brain gene expressions of neuronal activation markers (*Jun* and *JNK*) and lGnRH-I and -III, and lGnRH peptide concentrations in the forebrain, hindbrain, and plasma. The results confirmed that waterborne 3kPZS modulated the synthesis and release of lGnRH-I and -III in immature sea lamprey.

## Results

### Natural male pheromone mixtures facilitate sexual maturation

Exposure to spermiating male washings (SMW, natural male pheromone mixtures) facilitated reproductive maturation in immature males and females (χ^2^ = 5.042, dF = 1, p < 0.05; Table 1). On average, immature females exposed to SMW began to ovulate in 21 days, whereas immature females exposed to prespermiating male washings (PSMW, no pheromone control) did not start to ovulate until day 40. For immature males, the average time to spermiate when exposed to SMW was 17 days; in contrast, when exposed to PSMW, none of the immature males produced milt by day 40. Immature male lamprey appeared to mature faster (17 ± 3 days) than immature females (21 ± 8 days) after SMW exposure.

**Table 1:** Natural pheromones facilitate sexual maturation

**Treatment**	**Animals**	**Days to mature (Mean ± S.E.M)**	**Percentage of maturation**	**No. of animals (Immature/Mature)**
PSMW	POF	40	16.7	5/1
PSMW	PSM	ND	0	6/0
SMW	POF	21 ± 8	50	3/3
SMW	PSM	17 ± 3	50	3/3

Exposure to water conditioned by mature male sea lampreys (spermiating male washings, SMW) facilitated reproductive maturation in immature males and females. On average, immature females (POF) exposed to SMW began to ovulate in 21 days, whereas POF exposed to immature male washings (PSMW) did not ovulate until day 40. For immature males (PSM), the average time to spermiate after exposure to SMW was 17 days; in contrast, when exposed to PSMW, none of the PSMs had spermiated by day 40. χ^2^ = 5.042, dF = 1, p < 0.05 (Combine POF and PSM). ND: Not determined.

### Sex difference in steroid responses to natural male pheromone mixtures

**Figure 1:**
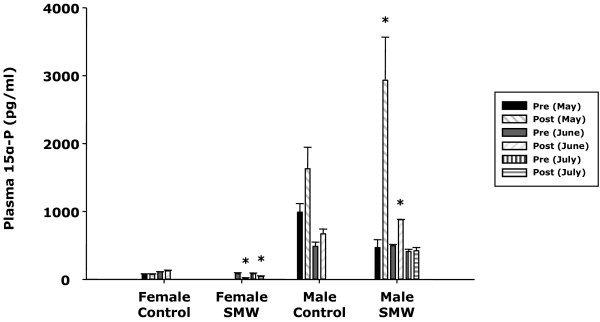
**Sex difference in seasonal steroidal responses after natural pheromone exposure in sea lamprey.** Exposure to mature male washings (SMW) for 24 h decreased plasma 15α-hydroxyprogesterone (15α-P) concentrations in immature females in June and July while the same treatment increased 15α-P concentrations in immature males in May and June. Lake Huron water (control) and immature male washings (data not shown) had no effect on 15α-P levels in either sex. Females did not have detectable 15α-P in their plasma early in the spawning season (May), but as the season progressed in June and July, plasma 15α-P concentrations became detectable. In immature males, changes in circulating 15α-P after SMW treatment was greater earlier in the spawning season (May) but diminished in later in the season (July). Sex differences in plasma 15α-P concentrations were significant (p < 0.05). * Statistically significant between pre- and post-treatment level in the same group (p < 0.05).

SMW exposure for 24 h altered plasma 15α-P concent-rations in immature sea lamprey (one-way ANOVA, p < 0.05; Figure 1). There were seasonal effects and sex differences in plasma 15α-P concentrations after exposure to SMW. Plasma 15α-P concentrations increased in immature males but decreased or showed no effect in immature females (Figure 1). Immature males showed an increase in 15α-P concentrations in response to SMW exposure early in the spawning season (May and June) while immature females did not show the decrease in response to SMW until mid-season (June) and the response dropped abruptly after July (Figure 1).

### Exposure to 3kPZS increased plasma 15α-P in males

**Figure 2:**
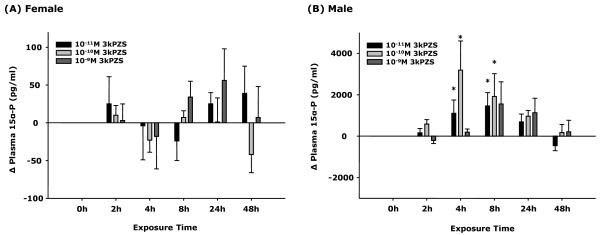
**Sex difference in steroidal responses after exposure to synthesized pheromone component in sea lamprey.** Exposure to 3kPZS increased plasma 15α-hydroxyprogesterone (15α-P) concentrations in immature male but not in immature female sea lamprey. Δ15α-P = (post-treatment 15α-P level) - (pre-treatment 15α-P level). * Statistically different from 0 h control group (p < 0.05).

Immature males showed elevated plasma 15α-P concent-rations after continuous exposure to a wide range of 3kPZS for 4 h up to 8 h (2- to 4-fold increase, Figure 2). Exposure to 10^-10^ M 3kPZS for 4 h was the most effective treatment to increase plasma 15α-P concentration (4-fold change, Figure 1). 3kPZS had no effect on plasma 15α-P concentrations in immature females (Figure 2). There was no apparent dose effect of 3kPZS within the range examined. In fact, higher concentration (10^-9^ M) of 3kPZS appeared to show longer latency in elevating plasma 15α-P compared to lower concentration (10^-11^ M or 10^-10^ M, Figure 2).

### Induction of forebrain gene expression after 3kPZS exposure in males

**Figure 3:**
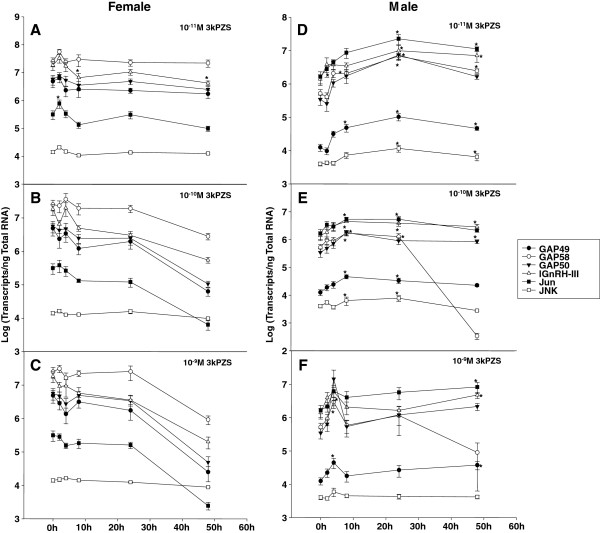
**Sex difference in forebrain gene expressions after exposure to synthesized pheromone component in sea lamprey.** Exposure to 10^-11^ M 3kPZS increased *Jun* mRNA (2 h, p < 0.05) but decreased lGnRH-III mRNA concentrations (8 h, 48 h, p < 0.05) in the forebrain of immature females (A-C). Exposure to 3kPZS increased GAP49, 50, 58, lGnRH-III, *Jun* and *JNK* mRNA concentrations in the forebrain of immature males (D-E). The following data points are statistically different from 0 h control (p < 0.05). D. GAP49: 8 h, 24 h, and 48 h; GAP58: 4 h, 8 h, 24 h & 48 h; GAP50: 24 h; lGnRH-III: 2 4 h & 48 h; *Jun*: 24 h & 48 h; *JNK*: 24 h & 48 h. E. GAP49: 8 h & 24 h; GAP58: 8 h & 24 h; GAP50: 8 h, 24 h & 48 h; lGnRH-III: 8 h & 24 h; *Jun*: 8 h & 24 h; *JNK*: 8 h & 24 h. F. GAP49: 4 h & 48 h; GAP58: 4 h; lGnRH-III: 4 h; Jun: 48 h.

3kPZS was most effective at 10^-11^ M with 24 h exposure time, inducing 7- to 21-fold increase in forebrain gene expressions. As the concentration of 3kPZS increased, the peak of gene expressions shifted to earlier time points (Figure 3) but the magnitude decreased.

Exposure to 10^-11^ M 3kPZS increased all mRNA transcripts examined in the forebrain of immature males (Figure 3). All three known splice variants of lGnRH-I mRNA were altered by 3kPZS exposure. GAP49 transcripts increased after exposure for 8 h (4-fold), 24 h (8-fold), and 48 h (4-fold). Increases in GAP58 transcripts occurred earlier and lasted longer after exposure for 4 h (4-fold), 8 h (4-fold), 24 h (13-fold), and 48 h (5-fold). GAP50 transcripts only increased after 24 h exposure (21-fold). lGnRH-III transcripts increased after exposure for 24 h (7-fold) and 48 h (5-fold). *Jun* transcripts increased after exposure for 8 h (5-fold), 24 h (14-fold), and 48 h (7-fold). *JNK* transcripts increased after exposure for 8 h (2-fold) and 24 h (3-fold).

Exposure to 10^-10^ M 3kPZS also increased all mRNA transcripts examined in the forebrain of immature males (Figure 3). GAP49 transcripts increased after exposure for 4 h (2-fold), 8 h (4-fold), and 24 h (3-fold). GAP58 transcripts increased after exposure for 8 h (3-fold) and 24 h (2-fold). GAP50 transcripts only increased after exposure for 8 h (5-fold). lGnRH-III transcripts increased after exposure for 8 h (3-fold), 24 h (3-fold), and 48 h (2-fold). Jun transcripts increased the earliest, after exposure for 2 h (2-fold), 8 h (3-fold), and 24 h (3-fold). *JNK* transcripts increased after exposure for 8 h (2-fold) and 24 h (2-fold).

Exposure to 10^-9^ M 3kPZS only increased lGnRH-I transcript variants, lGnRH-III and *Jun* mRNA concentrations in the forebrain of immature males (Figure 3). GAP49 transcripts increased after exposure for 4 h (2-fold), 8 h (4-fold), and 24 h (3-fold). GAP58 transcripts increased later after exposure for 8 h (3-fold) and 24 h (2-fold). GAP50 transcripts only increased after 8 h exposure (5-fold). lGnRH-III transcripts increased after exposure for 8 h (3-fold), 24 h (3-fold), and 48 h (2-fold). *Jun* transcripts rose the earliest, after exposure for 2 h (2-fold), 8 h (3-fold), and 24 h (3-fold).

### Sex difference in forebrain gene expression after 3kPZS exposure

Immature females showed increases in *Jun* (2-fold) and *JNK* (2-fold) expression rapidly (2 h) after 10^-11^ M 3kPZS exposure (Figure 3), whereas immature males had more delayed responses (≥ 4 h, Figure 3). Exposure to 10^-10^ M or 10^-9^ M 3kPZS had no effect on forebrain gene expression in immature females (Figure 3).

### Differential effect of 3kPZS on hindbrain gene expression

**Figure 4:**
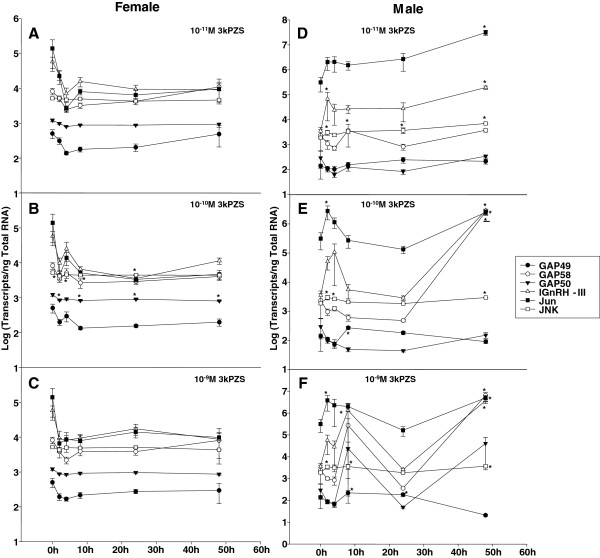
**Sex difference in hindbrain gene expressions after exposure to synthesized pheromone component in sea lamprey.** Exposure to 10^-10^ M 3kPZS decreased GAP50 mRNA (2 h, 8 h, 24 h, and 48 h, p < 0.05) and *JNK* mRNA (2 h, 4 h, 8 h, and 24 h, p < 0.05) concentrations in the brain stem of immature females (A-C). Exposure to 3kPZS increased GAP49 & 58, lGnRH-III, *Jun* and *JNK* mRNA concentrations in the brain stem of immature males (D-E). The following data points are statistically different from 0 h control (p < 0.05). D. lGnRH-III: 2 h & 48 h; *Jun*: 48 h; *JNK*: 2 h, 8 h, 24 h & 48 h. E. GAP58: 48 h; lGnRH-III: 48 h; *Jun*: 2 h & 48 h; *JNK*: 2 h, 4 h & 48 h. F. GAP49: 8 h & 24 h; GAP58: 48 h; lGnRH-III: 8 h & 48 h; *JNK*: 2 h & 8 h.

In the brain stem of immature males, 3kPZS seemed to be most effective at 10^-10^ M in increasing gene expressions, and the response appeared to be phasic with an earlier peak (2 h) and a delayed peak (48 h) at the time points examined. On the other hand, in the brain stem of immature females, only 10^-10^ M 3kPZS decreased GAP50 expression after 2 h (2-fold), 8 h (3-fold) and 48 h (2-fold) exposure (Figure 4). 3kPZS at other concentrations examined showed no effect on hindbrain gene expression in immature females (Figure 4).

The brain stem of immature males showed more pronounced gene expression changes than the forebrain after exposure to 10^-11^ M 3kPZS. lGnRH-III transcripts increased after 2 h (19-fold) and 48 h (55-fold) exposure (Figure 4). *Jun* transcripts increased after 48 h exposure (99-fold, Figure 4). *JNK* transcripts increased after 2 h (2-fold), 8 h (2-fold), 24 h (2-fold), and 48 h (4-fold) exposure (Figure 4).

At 10^-10^ M 3kPZS increased GAP49 transcripts after 8 h exposure (2-fold). *Jun* transcripts increased after 2 h (9-fold) and 48 h (8-fold) exposure. Prolonged exposure to 10^-10^ M 3kPZS (48 h) increased GAP58 (1122-fold), lGnRH-III (826-fold), and *JNK* (2-fold) transcripts in the brain stem of immature males (Figure 4).

At 10^-9^ M 3kPZS increased lGnRH-III transcripts after 8 h (379-fold) and 48 h (1052-fold) exposure (Figure 4). *Jun* transcripts increased after exposure for 2 h (12-fold) and 48 h (16-fold, Figure 4). *JNK* transcripts increased after exposure for 2 h (2-fold), 4 h (2-fold), 8 h (2-fold), and 48 h (2-fold, Figure 4). Prolonged exposure to 10^-9^ M 3kPZS (48 h) decreased GAP49 (7-fold) but increased GAP58 (2735-fold) expressions in the brain stem of immature males (Figure 4).

### Differential effect of 3kPZS on forebrain and plasma lGnRH peptide concentrations

**Figure 5:**
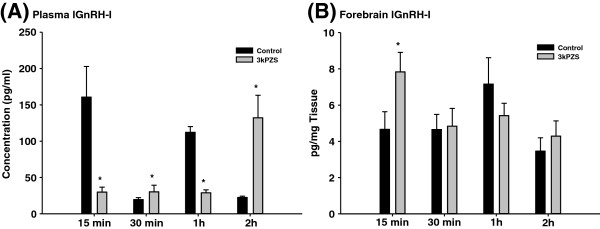
**Differential effects of synthesized pheromone component on plasma and forebrain lGnRH-I concentrations in male sea lamprey.** (**A**) Plasma lGnRH-I concentrations showed various effects after 10^-10^ M 3kPZS exposure. Exposure to 10^-10^ M 3kPZS increased lGnRH-I concentrations in the forebrain (**B**) but had no effect in the brain stem (data not shown).

**Figure 6:**
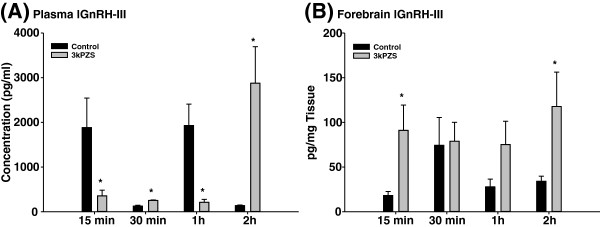
**Differential effects of synthesized pheromone component on plasma and forebrain lGnRH-III concentrations in male sea lamprey.** (**A**) Plasma lGnRH-III concentrations showed various effects after 10^-10^ M 3kPZS exposure. Exposure to 10^-10^ M 3kPZS increased lGnRH-III concentrations in the forebrain (**B**) but had no effect in the brain stem (data not shown).

Exposure to 10^-10^ M 3kPZS increased lGnRH-I and -III peptide concentrations in the forebrain (Figures 5 &6) but had no effect in the brain stem of immature males (Additional file 1: Figures S1& S2). Plasma lGnRH-I and -III peptide concentrations pulsed at the short time course after 3kPZS exposure. 3kPZS increased plasma lGnRH-I and -III peptide concentrations when the control level was low, but this effect was inhibited when the control level was high (Figures 5 &6).

### No effect of 3kPZS on the number of lGnRH neurons in the hypothalamus

The distribution of lGnRH mRNA, observed in the *in situ* hybridization experiments, was similar to that observed by Reed et al. [29], and was found mainly in the preoptic-hypothalamic area (Additional file 1: Figure S3). Immunocytochemistry using antibodies specific for lGnRH-I and -III demonstrated that the distributions of lGnRH-I or lGnRH-III immunoreactivities in lamprey brain (Additional file 1: Figures S4 & S5) were consistent with data described by Nozaki et al. [30]. Exposure to 3kPZS had no effect on the number of lGnRH-I or -III immunoreactive cells in the preoptic-hypothalamic area.

## Discussion

To our knowledge, this is the first study of the dose response and time course of the priming effects of a single pheromone component on GnRH synthesis and release. The synthesized male pheromone molecule affects both sexes but induces more dramatic responses in males. An interesting possibility is that immature males develop the ability to respond to 3kPZS as a result of chemical spying [31] since odors from conspecifics of the same sex tend to diminish reproductive activities in most species and there is no benefit to the mature males in broadcasting a signal to immature males [32]. As migratory adults arrive at the spawning ground with various sexual maturity at different times throughout the spawning season (about 2–3 months), spying on sex pheromones to synchronize gonadal development and other aspects of spawning readiness would be beneficial because sea lamprey are semelparous, i.e., spawning only once in their life time. This is especially important for immature males since they have to build nests and accelerate final sexual maturation so that they can release sex pheromones to attract mature females. The sex difference in the response to 3kPZS also revealed that different genders are likely tuned in to different chemical signals.

Waterborne 3kPZS increased forebrain lGnRH peptide concentrations and gene expressions, and altered plasma lGnRH peptide and sex steroid concentrations. These results indicate that 3kPZS modulates GnRH release, which is important for steroidogenesis and final sexual maturation. Mammalian neurosecretory cells in the hypothalamus display pulsatile activities [33], and ovulation is induced by a LH (one of the mammalian counter-parts of GTH) surge which is preceded by a surge of GnRH [LH releasing hormone (LHRH) in mammals] secretion into the brain portal system [34-36]. Parallel to this preovulatory increase in GnRH secretion is an increase in GnRH gene expression, as indicated by expression of the immediate-early genes (*c-fos* and *c-jun*) [37,38] and elevated GnRH mRNA [39,40], and increased levels of newly synthesized GnRH [41]. Lampreys and teleost fishes, however, lack the portal system in the brain, and GnRH neurons either terminate directly in the pituitary gland or release GnRH directly into the third ventricle [42,43]. Teleost fishes have well-characterized GTH with similar function and regulatory mechanisms as its mammalian counterpart [LH and follicle-stimulating hormone (FSH)]. A cDNA encoding lamprey GTH subunit β has been cloned, but its function has not been validated [44].

We found differential expression of lGnRH-I and -III transcripts in sea lamprey brain exposed to 3kPZS. Lampreys are the most primitive vertebrates for which multiple GnRH neurohormones are involved in pituitary-reproductive activity [45,46]. Both lGnRH-I and -III have been shown to induce gonadal maturation, steroidogenesis, and spermiation or ovulation in adult sea lamprey [45-49]. In immunocytochemical studies, both immunoreactive-lGnRH-I and -III can be found in the cell bodies of the rostral hypothalamus and preoptic area in larval and adult sea lamprey [30,44,50,51]. lGnRH-III was considered the more active form during development and gonadal maturation [52]. Our results indicated that lGnRH-III concentrations were more responsive to pheromone exposure. However, lGnRH-I may be more important in inducing behavioral responses in mature females, given that the transcripts of its three splice variants fluctuated in the brainstem after pheromone exposure while lGnRH-III transcripts stayed constant.

It is interesting that three lGnRH-I splice variants also showed differential expressions after pheromone exposure. Lamprey GnRH-I precursor was the first identified agnathan GnRH to contain the same tripartite structure with the signal peptide, GnRH decapeptide, and GnRH-associated peptide (GAP) as gnathostome GnRH precursors [52]. Unlike other known vertebrate GnRH precursors, which typically have one or two splice variants, three distinct splice variants were isolated and sequenced in lampreys [52]. The lGnRH-I splice variants, GAP49, GAP50 and GAP58, differed in the length of the GAP coding sequence [52]. The relative abundance of these splice variants in our results followed a magnitude decrease in the order of GAP58, GAP50, and GAP49. According to the changes after pheromone exposure, GAP50 seemed to be important for forebrain function whereas GAP49 and 58 may be associated with brainstem function in both sexes. However, forebrain GAP49 and brainstem GAP50 may serve additional functions in immature females [53].

Corresponding to its gene expression level in male sea lamprey, lGnRH-III is the most prominent lGnRH peptides in the brain and plasma. Its concentration is around 20 times more than lGnRH-I. We also found that 3kPZS altered the pulsatile pattern of GnRH concentrations in plasma. Pulsatile release of GnRH is a common neurobiological feature in vertebrates. It is autonomous within the GnRH neuronal network without spontaneous pacemaker cells [54]. It is interesting that menstrual synchrony in humans is mediated by pheromones through two opposing effects of the same compounds [55]. Axillary compounds (putative human pheromones) from donor women in the follicular phase shortened both the time to ovulation and the length of the menstrual cycle in recipients and those in the ovulatory phase delayed ovulation and lengthened the total cycle [55]. We found that when GnRH level is low in the control group, 3kPZS increased GnRH peptide concentrations in the plasma whereas when GnRH level is high in the control group, 3kPZS showed opposite effects. There may be some similarity in the control mechanism involved in this process.

Pulsatile GnRH regulates the gonadotropin subunit genes in a differential manner, with faster frequencies (8 to 60 min pulse intervals) favoring mammalian LH β subunit gene expression and slower frequencies (≥ 120 min pulse intervals) favoring the expression of FSH β subunit [56]. The mechanism is through the activation of GnRH receptor and its signaling cascades including JNK and its substrate JUN [57-61]. We found that 3kPZS induced GnRH release within 15 min, and *Jun* and *JNK* gene expression after 2 h exposure, suggesting that 3kPZS can modulate GnRH release and its signaling cascades.

The detection of lGnRH peptides in the plasma was a surprise since many previous attempts using HPLC and radioimmunoassays had failed to detect lGnRH peptides in sea lamprey plasma [62-65]. However, high-affinity binding sites for lGnRH peptides were detected in lamprey gonads, and GnRH appears to stimulate steroidogenesis independent of the pituitary [48,66]. Previous reports also noticed that the concentrations of brain GnRH peptides in lamprey occur at higher concentrations than seen in other vertebrates [62,67], suggesting that there may be a higher production or metabolism (or both) of each of the lGnRH peptides compared to gnathostomes [65]. Taken together, lGnRH peptides likely serve as hormones that are released into the third ventricle and then into the blood stream, and act on target organ such as gonads. Therefore, our results provide an additional explanation for GnRH binding affinity in peripheral organs [66]. Furthermore, our results support a possible peripheral endocrine function of lGnRH peptides, which may bypass the pituitary gland in the HPG axis.

**Figure 7:**
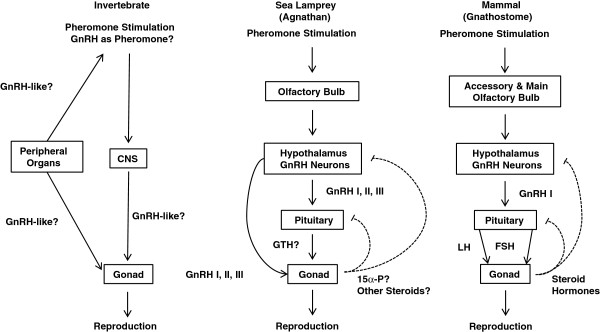
A schematic diagram of the evolutionary transition between pheromone and GnRH control from invertebrates to sea lamprey (agnathan) to mammals (gnathostome).

The acquisition of a HPG axis was a seminal event in vertebrate evolution leading to the neuroendocrine control of many biological functions [68]. GnRH is a critical neuropeptide that acts through the HPG axis to regulate vertebrate reproduction. However, invertebrate GnRH peptides seem to exert a wide range of central and peripheral functions not limited to reproduction [69]. In addition, GnRH may even function as spawning pheromone in invertebrates [70]. The presence of lGnRH peptides in sea lamprey plasma indicates that the function of lGnRH peptides may not be fully centralized. It is likely that sea lamprey GnRH peptides function in an evolutionary transitional state where they not only act on the HPG axis, but also exert hormonal functions through systemic circulation. Furthermore, environmental factors such as a single pheromone component (i.e. 3kPZS) can modulate the HPG axis through olfaction. The considerable specialization of GnRH in the HPG axis as a reproductive activator may be a phenomenon specific to jawed vertebrates (gnathostomes) (Figure 7).

## Conclusions

3kZPS is a primer pheromone in sea lamprey that exerts its functions through the HPG axis via GnRH release.

## Methods

### Animals

Sea lamprey were collected by agents of the US Fish and Wildlife Service Marquette Biological Station (Marquette, MI) and Department of Fisheries and Oceans Canada Sea Lamprey Control Centre (Sault Ste. Marie, ON). Pheromone treatments of animals were conducted at the US Geological Survey Great Lakes Science Center Hammond Bay Biological Station (Millersburg, MI). For each set of experiments, all test subjects were captured from the same stream on the same day to reduce variation in maturity. Standard operating procedures for transporting, maintaining, handling, anesthetizing, and euthanizing sea lamprey were approved by the Institutional Committee on Animal Use and Care of Michigan State University (AUF#05/09-088-00). Ethical guidelines were followed throughout the course of this research. Pheromone exposure time in the following experiments was chosen according to our previous publications [14,53,71,72].

#### Pheromone exposure experiment 1

Twelve immature males were held in separate tanks (100 gallon; 16°C; water replenishment 1 L min^-1^), and acclimated for 2 days. 16°C was within the optimal temperature range for lamprey reproduction [73]. Animals were randomly assigned to two treatment groups (6/group): water from a tank (pheromone source tank) containing 5 mature males (spermiating male washings, SMW) or 5 immature males (prespermiating male washings, PSMW). Every two days, animals were checked for spermiation. The number of days to reach spermiation was recorded. The same experiment was repeated using 12 immature females (checking for egg release), and data were analyzed with the χ^2^ test.

#### Pheromone exposure experiment 2

Two hundred and fifty-two immature females and males were acclimated as described in experiment 1, and assigned to two treatment groups (control or SMW). At 0 h (immediately before introducing the odorants) and 24 h (completion of odorant treatment), 1 ml of blood was drawn to obtain plasma according to previously established protocols and stored at −80°C until use [71,72]. Plasma samples were assayed for immunoreactive 15α-P using HPLC and radioimmunoassays [71,72]. The same experiment was repeated in the summers of 2001, 2002 and 2003. Data were pooled for each month (May, June and July) and analyzed by One-way ANOVA for repeated measurements (before and after pheromone exposures). If treatment effects were detected (p < 0.05), Fisher’s PLSD *post hoc* tests were followed to determine which treatment induced apparent changes in the parameters measured.

#### Pheromone exposure experiment 3

To examine the time course of hormonal responses of immature sea lamprey exposed to 3kPZS at a wide range of concentrations, 108 immature males were acclimated for 2 days (6/tank; test tank: 200 L, 16°C, water replenishment 1 L min^-1^). Each tank was randomly assigned a treatment of 10^-11^ M, 10^-10^ M or 10^-9^ M 3kPZS. Immediately before and after pheromone exposure, 1 ml blood was drawn for plasma 15α-P analyses [71,72]. One-way ANOVA was used to compare the changes in plasma 15α-P concentration [Δ15α-P = (post-treatment 15α-P level) - (pre-treatment 15α-P level)] among groups treated with the same pheromone concentration but sampled at different time points. Fisher’s PLSD post hoc tests were performed if the ANOVA showed significant effects (p < 0.05).

At 0 (control), 2, 4, 8, 24 and 48 h after pheromone exposure, animals were euthanized with 0.5% MS222 (Sigma, St. Louis, MO). Eight brain samples were snap frozen and analyzed for transcripts of lGnRH-I (GAP49, GAP50, and GAP58) [74] and -III, *jun*, *JNK*, and 40S ribosomal RNA. Six brain samples from each group were fixed in 4% paraformaldehyde (in 0.1 M phosphate buffer, pH 7.4) for immunocytochemistry (ICC) and *in situ* hybridization (ISH) analyses. From each preserved brain, serial transverse sections of 20 μm were collected. Neighboring sections were processed for lGnRH-I or -III ICC [53], or lGnRH mRNA ISH. Negative controls (deprived of primary antibody for ICC or using sense probe for ISH) were processed simultaneously in each experiment. ISH and ICC results were examined by investigators with no knowledge of the experimental condition. The number of immunoreactive cells in the preoptic-hypothalamic area was counted [53], and the average number in a square area (0.0625 mm^2^) was used for statistical analyses. Brain nuclei were identified according to sea lamprey brain atlas by Nieuwenhuys and Nicholson [75]. The nonparametric Kruskal-Wallis test was used to compare the number of lGnRH-I or -III immunoreactive neurons among treatment groups. Parallel experiments were conducted with immature females.

#### Pheromone exposure experiment 4

Since 3kPZS had no dramatic effects in immature females and the gene expression changed after 2 h exposure, we only examined the time course of GnRH release in immature males exposed to vehicle (5 ppm methanol) or 10^-10^ M 3kPZS. 64 immature males were acclimated as described above. Immediately and at 15 min, 30 min, 1 h or 2 h after pheromone exposure, animals were euthanized and blood was drawn by cardiac puncture for plasma lGnRH-I and -III analyses using UPLC-MS/MS (method described below). Brain samples were snap-frozen and analyzed for lGnRH-I and -III using ultra performance liquid chromatography coupled with tandem mass spectrometry (UPLC-MS/MS).

***In situ Hybridization*** used digoxigenin-labeled antisense and sense RNA probes generated by *in vitro* Transcription Systems (Promega BioSciences, San Luis Obispo, CA, USA). Sections were treated with proteinase K at room temperature (R.T.) for 10 min followed by 4% paraformaldehyde (0.1 M phosphate buffer, pH 7.4) for 15 min. Sections were incubated in prehybridization solution [50% formamide, hybridization salt (150 mM NaCl, 50 mM EDTA, 50 mM PIPES), Denhardt’s solution (Sigma, St. Louis, MO, USA), 2.5 μg/ml calf thymus DNA, 2.5 μg/ml poly-adenosine, 0.2% sodium dodecyl sulfate (SDS), and 0.1% diethylpyrocarbonate] at 42°C for 2 h, and then in antisense or sense RNA probe in hybridization solution (prehybridization solution with 5% dextran sulfate) at 60°C overnight (about 18 h). Sections were rinsed with 4× SSC (150 mM NaCl and 15 mM sodium citrate), followed by 2× SSC (with 0.3% Tween-20) at 68°C for 15 min 3 times, 0.2× SSC (with 0.3% Tween-20) at 68°C for 15 min 3 times, 0.1× SSC (with 0.3% Tween-20) at R.T. for 15 min, and then 0.1 M phosphate buffer saline (PBS, pH 7.4). Sections were incubated in alkaline phosphatase-conjugated sheep-anti-digoxigenin Fab fragments (1:1000; Roche Applied Science, Indianapolis, IN, USA) with normal sheep serum (in 0.1 M PBS with 0.3% Tween-20) at 5°C overnight (about 18 h). Sections were rinsed in 0.1 M Tris buffer saline (TBS, pH 9.5) for 15 min 3 times, followed by nitroblue tetrazolium chloride and 5-bromo-4-chloro-3 indolyl phosphate substrate (Roche) for 1 h, rinsed in PBS (pH 7.4) for 15 min 3 times, counter stained with Nuclear Fast Red (Vector Laboratories, Inc., Burlingame, CA, USA) for 5 min, and coverslipped with Vector AQ mounting media.

***Real time quantitative RTQ-PCR*** followed the procedure described by Chung-Davidson et al. [53]. Synthetic oligos were used as standards and run on the sample plate. 40S ribosomal RNA was used as an internal standard and confirmed to have no change in the expression level in all experiments. RTQ-PCR data (among groups treated with same pheromone concentration but sampled at different time points) were analyzed by one-way ANOVA followed by Fisher’s PLSD post hoc tests if the ANOVA showed significant time effects (p < 0.05). The sequence for primers and TaqMan RGB probe (Applied Biosystems) for each mRNA was listed below. 40S ribosomal RNA: 5' primer (5'ACCTACGCAGGAACAGCTATGAC3'), probe (5'ATCTCGAGCAGCTGAA3'), 3' primer (5'CGACGAATTCCACCACATTG3'). Jun: 5' primer (5'CATGGCCGCAAACTTTGG3'), probe (5'CACGAACCTGACCAGC3'), 3' primer (5'CCACCTCCCTGCTGATGCT3'). JNK: 5' primer (5'TCAGGCGTGTGGCCAAGT3'), probe (5'CCATGACTTGATCGAATGT3'), 3' primer (5'GAATCAAATTGAGAACGCAAACG3'). Lamprey GnRH-III: 5' primer (5'TGACACGAACCCTGTCAATGA3'), probe (5'ATGCCCTCGCTGTGGT3'), 3' primer (5'ACAAAGGGTCTAAGAGACGTCACA3'). To analyze three transcripts of lGnRH-I, we used the same 5' primer (5'TGAATTACGCGCAGCACTACTC3') and probe (5'TGGAATGGAAACCCGG3'), but the 3' primers were designed so that they were located at the splice junction of each corresponding transcript (GAP49: 5'CTCCTCCAGGTCTCGTTTGC3'; GAP50: 5'CTCCTGCTCCAGGTCTCGTT3'; GAP58: 5'CCAGCTCTCGTGTGTGACTGA3').

### UPLC-MS/MS analyses of lGnRH

lGnRH-I standard was custom synthesized from GenScript USA Inc. (Piscataway, NJ, USA). lGnRH-III and LHRH were purchased from BAChem Americas, Inc. (Torrance, CA, USA). Each compound was dissolved in 50% methanol/H_2_O (v/v) to make 1 mg/ml stock solution, and stored at −20°C until use. Subsequent dilutions were made in 50% methanol/H_2_O. Calibration standard spiking solutions were prepared in a range from 0.01 to 10 ng/ml by spiking appropriate stock solutions to brain tissue or plasma extracts. Internal standard (LHRH) solution (50 ng/ml) was prepared in 50% methanol/H_2_O and 20 μl was added to each sample (1 ng per sample).

Brain samples were weighed and homogenized with 400 μl of 1% formic acid in cold (−20°C) acetonitrile with 1 ng internal standard. 1 ml of 1% formic acid in cold (−20°C) acetonitrile was added to the homogenate, incubated at −20°C for 15 min, and centrifuged at 15,800 × g for 20 min at 4°C. The supernatant was transferred to a new tube, freeze-dried using a CentriVap Cold Trap Concentrator (Labconco Co., Kansas City, MO, USA), and reconstituted in 1 ml water solution with 3% acetic acid and 1% TFA. 500 μl plasma samples were processed in a similar procedure without the homogenization step. The reconstituted supernatant was transferred to a SPE HLB cartridge preconditioned with 3 ml methanol followed by 3 ml loading solution (water/acetic acid/TFA, 96:3:1, v/v), washed with 3 ml loading solution and followed by 3 ml loading solution/methanol (70:30, v/v). Samples were eluted with 3 ml 3% acetic acid/ solution: methanol (30:70, v/v), freeze-dried and reconstituted in 100 μl 3% acetic acid/methanol (50:50, v/v), vortexed for 30 seconds and transferred to glass autosampler vials for UPLC-MS/MS analyses.

A Waters Xevo TQ-S mass spectrometry was coupled to an H-Class UPLC system with a Waters BEH C18 column (1.0 × 50 mm, 1.7 μm particle size) and oven temperature at 35°C. The injection volume was 10 μl, and the UPLC flow rate is 0.15 ml/min with a gradient (mobile phase A: 0.1% formic acid in water; B: 0.1% formic acid in acentonitrile): initial, 88% A and 12% B; 0.5 min 88% A, 4 min 65% A, 7 min 1% A, 8 min 1% A, 8 min 88% A, and 9 min 88% A. Mass spectra were acquired using electrospray ionization in positive ion mode and MRM. The capillary voltage, cone voltage, and rf lens setting were 3.20 kV, 64 V, and 0.3, respectively. The flow rates of cone gas and desolvation gas were 20 and 400 L/h, respectively. The source temperature and desolvation temperature were 150 and 500°C. Collision-induced dissociation employed argon as collision gas at a manifold pressure of 2 × 10^-3^ mbar, and collision energies and source cone potentials were optimized for each transition using Waters QuanOptimize software. Data were acquired with MassLynx 4.1 and QuanLynx softwares.

## Abbreviations

HPG: Hypothalamic-pituitary-gonadal; GnRH: Gonadotropin-releasing hormone; 3kPZS: 3-keto petromyzonol sulfate; 15α-P: 15α-hydroxyprogesterone; lGnRH-I: lamprey GnRH-I; lGnRH-III: lamprey GnRH-III; JNK: Jun N-terminal kinase; LH: Luteinizing hormone; GTH: Gonadotropin; SMW: Spermiating male washing; PSMW: Prespermiating male washing; LHRH: Luteinizing hormone-releasing hormone; FSH: Follicle-stimulating hormone; ICC: Immunocytochemistry; ISH: *In situ* hybridization; RTQ-PCR: Real-time quantitative PCR; UPLC-MS/MS: Ultra-performance liquid chromatography coupled with tandem mass spectrometry.

## Competing interests

The authors declare that they have no competing interests.

## Authors’ contributions

YWCD designed the study, carried out the sample collection and all experimental studies, performed the statistical analyses and wrote the manuscript. H Wang developed UPLC-MS/MS methods and collected data in Pheromone Exposure Experiment 4. MJS participated in Pheromone Exposure Experiment 1 and 2. MBB participated in Pheromone Exposure Experiment 2 and 3. NSJ and H Wu participated in Pheromone Exposure Experiment 3. WL conceived of the study, participated in its design and coordination and helped to write the manuscript. All authors read and approved the final manuscript.

## Supplementary Material

Additional file 1: Figure 1 3kPZS exposure had no effect on hindbrain lGnRH-I concentrations in immature male sea lamprey. Data are presented as mean ± S.E.M. **Figure 2.** 3kPZS exposure had no effect on hindbrain lGnRH-III concentrations in immature male sea lamprey. Data are presented as mean ± S.E.M. **Figure 3.** 3kPZS exposure did not change the number of lamprey (l) GnRH-positive neurons in the hypothalamus. Lamprey (l) GnRH *in situ* hybridization (ISH) showed positive cells (blue stain) in the preoptic area of immature female and male sea lamprey. 20 μm Transverse sections were counterstained with nuclear fast red (pink stain). Scale bar: 20 μm. 3 V: third ventricle. **Figure 4.** 3kPZS exposure did not change the number of lamprey (l) GnRH-Iimmunoreactive neurons in the hypothalamus. lGnRH-I-immunoreactive neurons (red stain) are located in the preoptic area of immature female and male sea lamprey. 20 μm transverse sections were counterstained with hematoxylin (blue/purple stain). Scale bar: 50 μm. 3 V: third ventricle. **Figure 5.** 3kPZS exposure did not change the number of lamprey (l) GnRH-IIIimmunoreactive neurons in the hypothalamus. lGnRH-III-immunoreactive neurons (red stain) are located in the preoptic area of immature female and male sea lamprey. 20 μm transverse sections were counterstained with hematoxylin (blue/purple stain). Scale bar: 50 μm. 3 V: third ventricle. Click here for file
