# Supplementary material for: Pheromonal bile acid 3-ketopetromyzonol sulfate primes the neuroendocrine system in sea lamprey
Source: BMC Neurosci. 2013 Jan 20;14:11. doi: 10.1186/1471-2202-14-11 (PMC3599739; doi:10.1186/1471-2202-14-11)

Suppl. Fig. 1. 3kPZS exposure had no effect on hindbrain lGnRH-I concentrations in immature male sea lamprey. Data are presented as mean ± S.E.M.

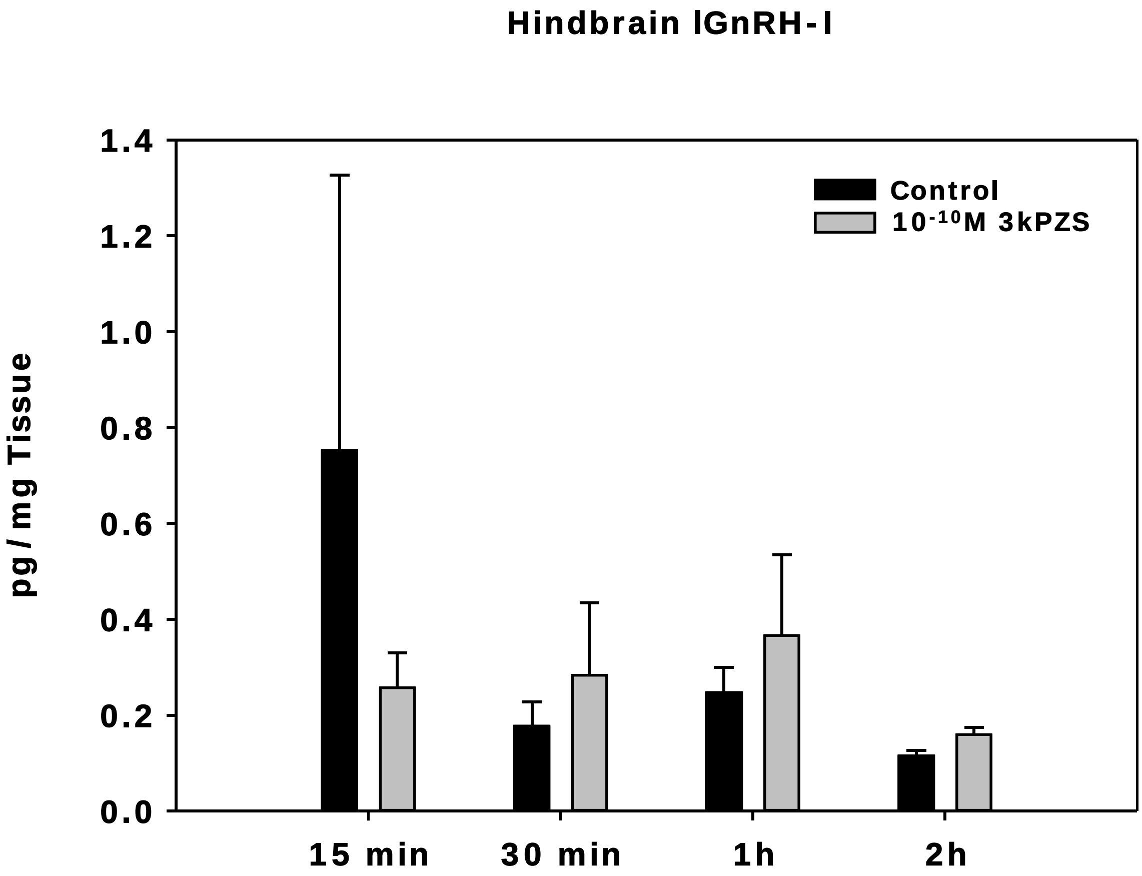

Supplement Fig. 2. 3kPZS exposure had no effect on hindbrain lGnRH-III concentrations in immature male sea lamprey. Data are presented as mean ± S.E.M.

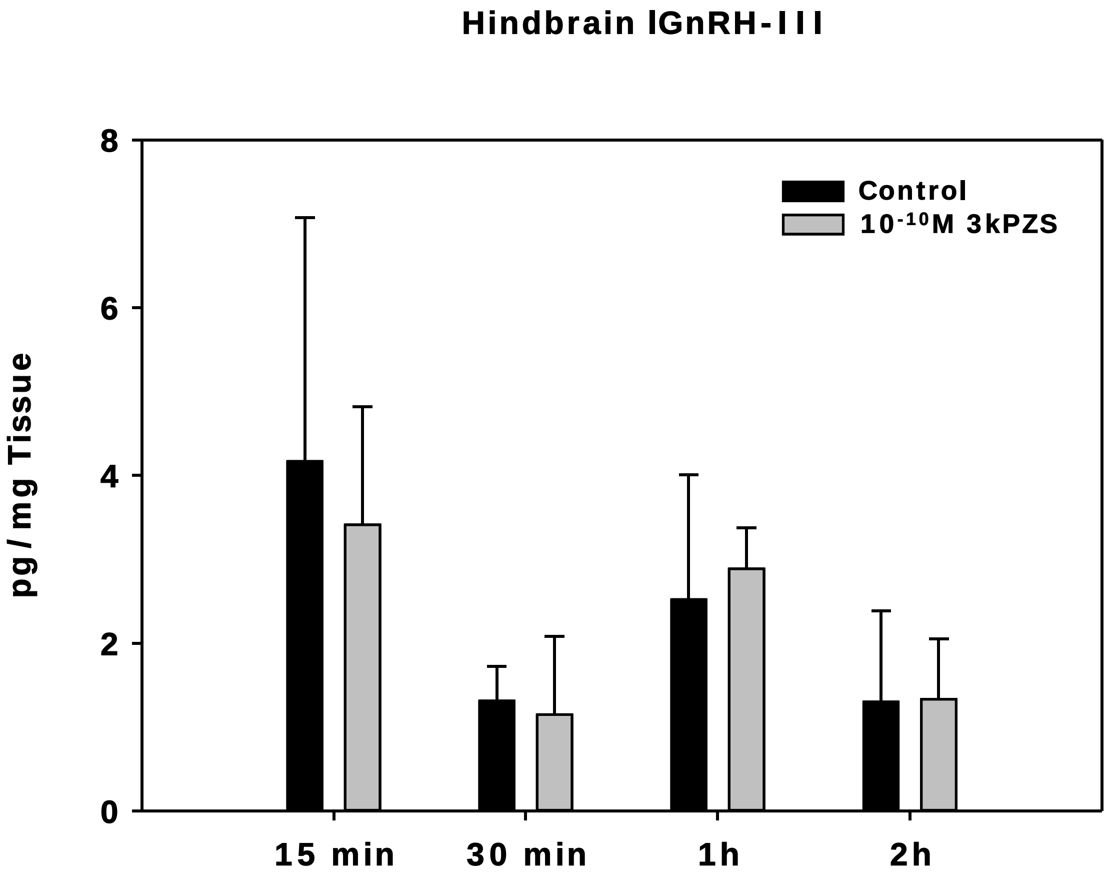

Suppl. Fig. 3. 3kPZS exposure did not change the number of lamprey (l) GnRH-positive neurons in the hypothalamus. Lamprey (l) GnRH *in situ* hybridization (ISH) showed positive cells (blue stain) in the preoptic area of immature female and male sea lamprey. 20µm Transverse sections were counterstained with nuclear fast red (pink stain). Scale bar: 20 µm. 3V: third ventricle.

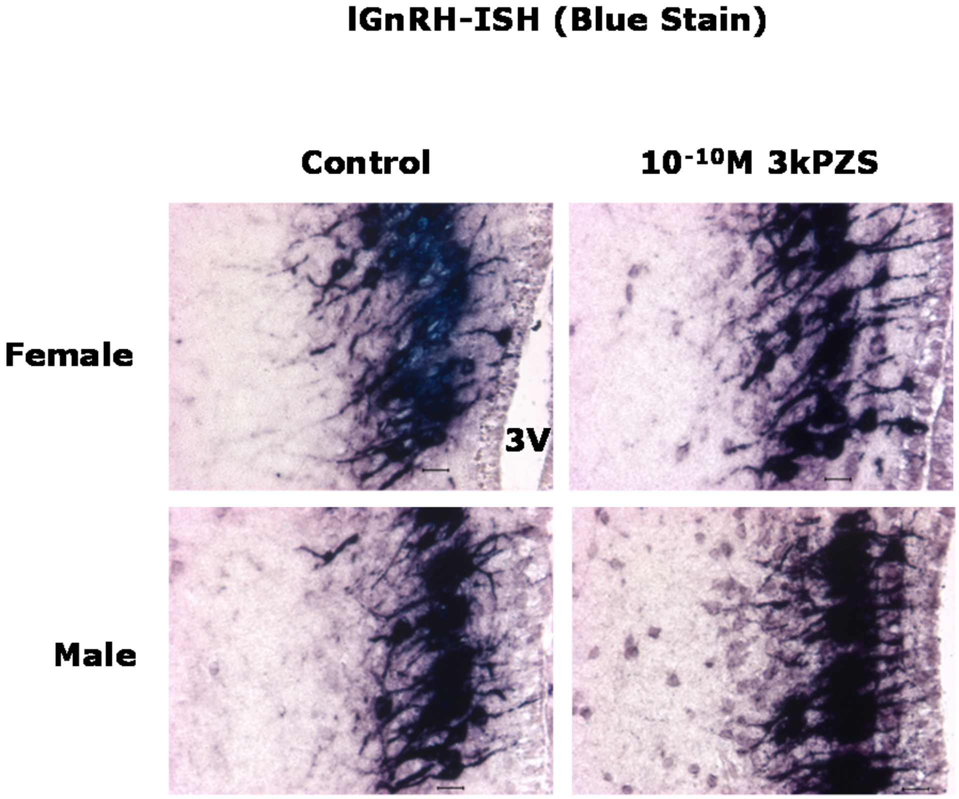

Suppl. Fig. 4. 3kPZS exposure did not change the number of lamprey (l) GnRH-I-immunoreactive neurons in the hypothalamus. lGnRH-I-immunoreactive neurons (red stain) are located in the preoptic area of immature female and male sea lamprey. 20 µm transverse sections were counterstained with hematoxylin (blue/purple stain). Scale bar: 50 µm. 3V: third ventricle.

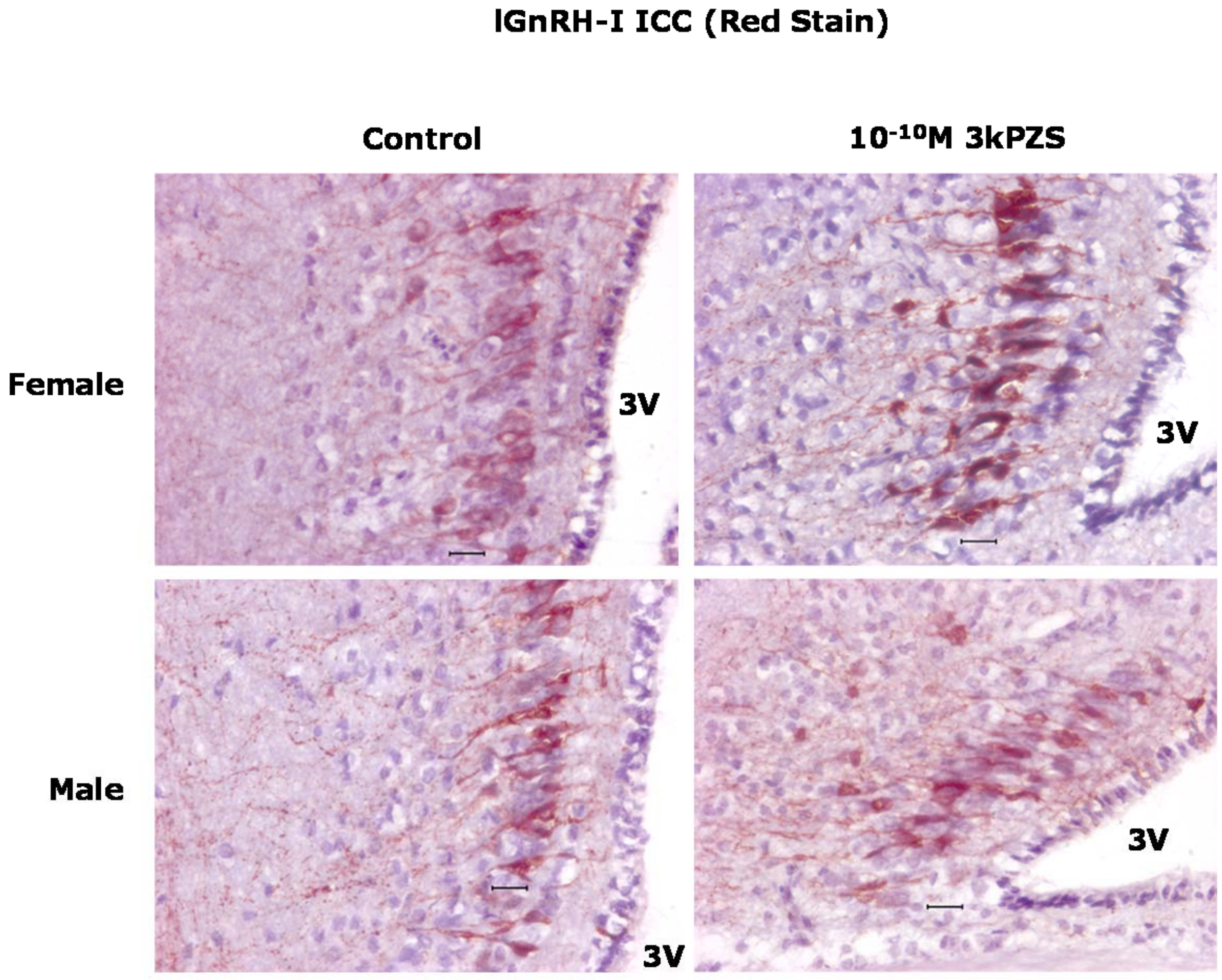

Suppl. Fig. 5. 3kPZS exposure did not change the number of lamprey (l) GnRH-III-immunoreactive neurons in the hypothalamus. lGnRH-III-immunoreactive neurons (red stain) are located in the preoptic area of immature female and male sea lamprey. 20 µm transverse sections were counterstained with hematoxylin (blue/purple stain). Scale bar: 50 µm. 3V: third ventricle.

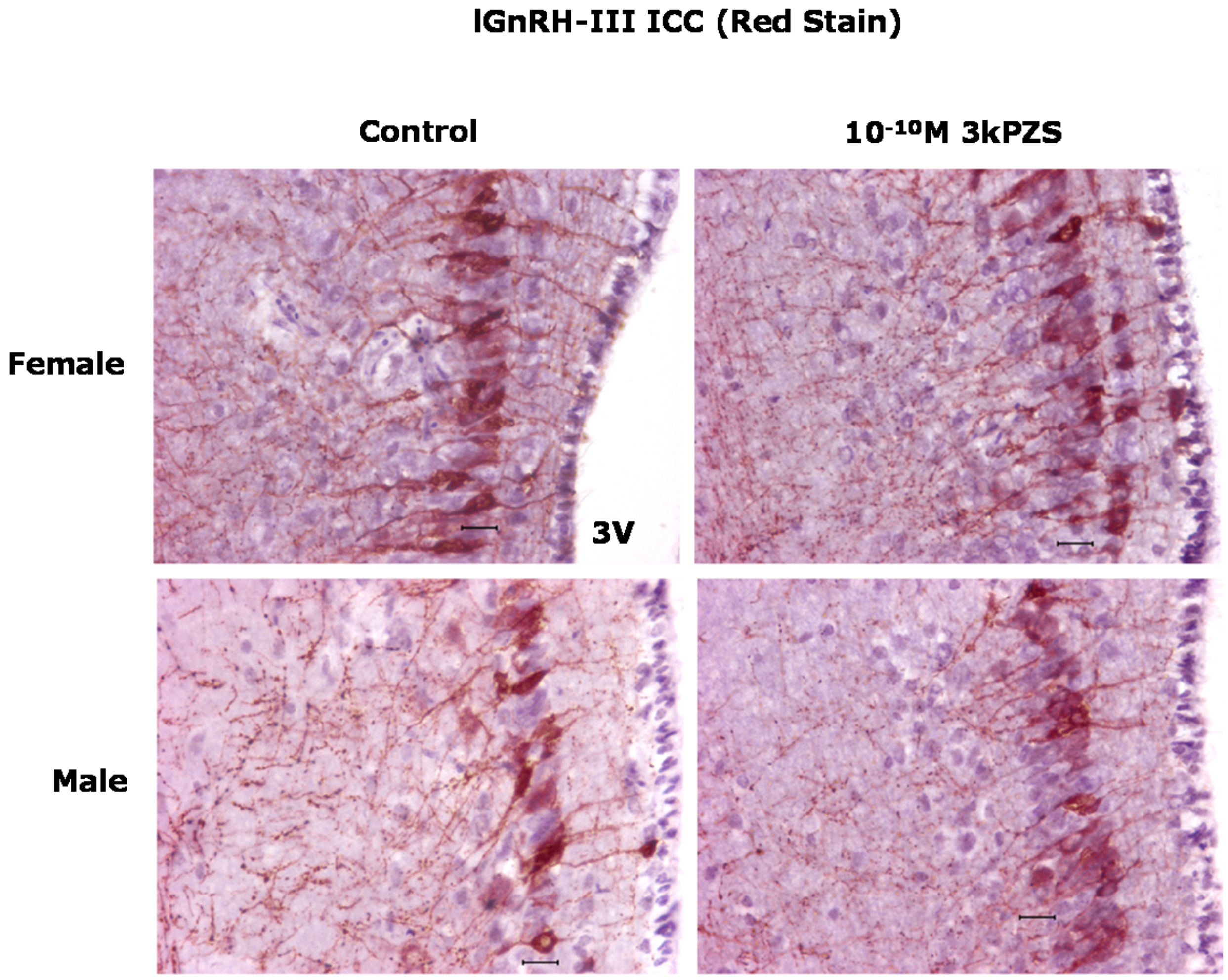

Supplement: Additional file 1: Figure 1 — 3kPZS exposure had no effect on hindbrain lGnRH-I concentrations in immature male sea lamprey. Data are presented as mean ± S.E.M. Figure 2. 3kPZS exposure had no effect on hindbrain lGnRH-III concentrations in immature male sea lamprey. Data are presented as mean ± S.E.M. Figure 3. 3kPZS exposure did not change the number of lamprey (l) GnRH-positive neurons in the hypothalamus. Lamprey (l) GnRH in situ hybridization (ISH) showed positive cells (blue stain) in the preoptic area of immature female and male sea lamprey. 20 μm Transverse sections were counterstained with nuclear fast red (pink stain). Scale bar: 20 μm. 3 V: third ventricle. Figure 4. 3kPZS exposure did not change the number of lamprey (l) GnRH-Iimmunoreactive neurons in the hypothalamus. lGnRH-I-immunoreactive neurons (red stain) are located in the preoptic area of immature female and male sea lamprey. 20 μm transverse sections were counterstained with hematoxylin (blue/purple stain). Scale bar: 50 μm. 3 V: third ventricle. Figure 5. 3kPZS exposure did not change the number of lamprey (l) GnRH-IIIimmunoreactive neurons in the hypothalamus. lGnRH-III-immunoreactive neurons (red stain) are located in the preoptic area of immature female and male sea lamprey. 20 μm transverse sections were counterstained with hematoxylin (blue/purple stain). Scale bar: 50 μm. 3 V: third ventricle. [file 1471-2202-14-11-S1.pdf]
